# The Synaptic Clock: SynGAP1 as a Molecular Timer of Postsynaptic Density Consolidation

**DOI:** 10.3390/biom16060876

**Published:** 2026-06-15

**Authors:** Zixuan Cao, Yibin Jia, Zhuoyuan Zhang, Hanjiang Xue, Hanwei Yu, Xin Li, Peng Luo

**Affiliations:** 1 Department of Neurosurgery, Xijing Hospital, Fourth Military Medical University, Xi’an 710032, China; zixuancao2022@163.com (Z.C.); jyb3142126@126.com (Y.J.); zzy990826@126.com (Z.Z.); xuehanjiang0203@163.com (H.X.); Y1744697589@163.com (H.Y.); 2 The Second Regiment, School of Basic Medicine, Fourth Military Medical University, Xi’an 710032, China; 3 Department of Anesthesiology, Xijing Hospital, Fourth Military Medical University, Xi’an 710032, China

**Keywords:** SynGAP1, liquid–liquid phase separation, critical period, gene therapy, postsynaptic density, isoform switching

## Abstract

*SYNGAP1*-related intellectual disability presents a therapeutic paradox where genetic rescue is highly effective in neonates but limited in adults, suggesting that deficiency represents a developmental trajectory violation rather than a static biochemical defect. By synthesizing molecular, biophysical, and clinical evidence, this review proposes the “Synaptic Clock” framework, redefining SynGAP1 as a critical developmental regulator. We hypothesize that SynGAP1 operates through a strictly ordered temporal sequence: Phase I (Scaffold Assembly) utilizes the α1 isoform and phase separation to establish the structural postsynaptic density, while Phase II (Catalytic Refinement) involves isoform switching to enable activity-dependent plasticity and homeostatic scaling. This model characterizes synaptic maturation as a biophysical transition from a fluid scaffold to a consolidated gel, potentially marking the biological closure of structural rescue windows. Based on this hypothesized temporal mapping, we establish a phase-stratified therapeutic roadmap—transitioning from early-stage “reset” strategies like gene replacement to late-stage “refinement” and “compensation” via pharmacological and neuromodulatory interventions. Ultimately, validating phase-specific biomarkers, including gamma oscillations and isoform stoichiometry, is essential for shifting from generic interventions toward precision, phase-matched medicine for neurodevelopmental timing.

## 1. Introduction

Biological systems are governed by temporal mechanisms that strictly order events to ensure critical interactions occur within defined windows of opportunity. At the synapse, the postsynaptic density (PSD) forms a specialized electron-dense region on the dendritic spine, composed of an intricate network of scaffold proteins, primarily accumulating discs large (DLG) family members like PSD-95 first, and neurotransmitter receptors, such as α-amino-3-hydroxy-5-methyl-4-isoxazolepropionic acid (AMPA) and *N*-methyl-D-aspartate (NMDA) receptors. This structure is essential for receiving and translating chemical signals into electrical responses. Drawing on foundational studies of PSD dynamics [1,2,3,4,5], we herein propose the “Synaptic Clock” as a conceptual framework. Extrapolating primarily from in vitro biophysical models, this framework describes an intrinsic molecular timer that is hypothesized to drive the PSD from a dynamic, fluid state in early development to a structurally consolidated architecture in maturity.

The *SYNGAP1* gene (encoding the SynGAP1 protein) serves as a key temporal regulator of this process. Mutations or haploinsufficiency of the *SYNGAP1* gene cause *SYNGAP1*-related intellectual disability (SRID), a paradigmatic disorder of disrupted synaptic timing characterized by global developmental delay, generalized epilepsy, and autism spectrum disorder (ASD) features [6,7,8,9,10,11,12]. Throughout this manuscript, *SYNGAP1* refers to the genetic locus and its transcription, while SynGAP1 denotes the multidomain Ras/Rap GTPase-activating protein (GAP) that participates in these developmental programs.

A central paradox currently confronts the field: in rodent models, early postnatal (P0–P14) genetic reinstatement of *SYNGAP1* yields profound rescue of cognitive and structural phenotypes. While genetic restoration in adulthood continues to offer beneficial functional and seizure-suppressive effects, the capacity to fully reverse established structural network deficits appears more restricted [13,14,15]. This decline indicates that SynGAP1 deficiency represents a developmental trajectory violation. Notably, protein-restoring therapies, such as antisense oligonucleotides (ASOs), are rapidly advancing toward clinical trials across diverse age groups, making it critical to understand how baseline synaptic maturity influences therapeutic outcomes.

### 1.1. SynGAP1: A Multidomain Key Regulator

**Figure 1:**
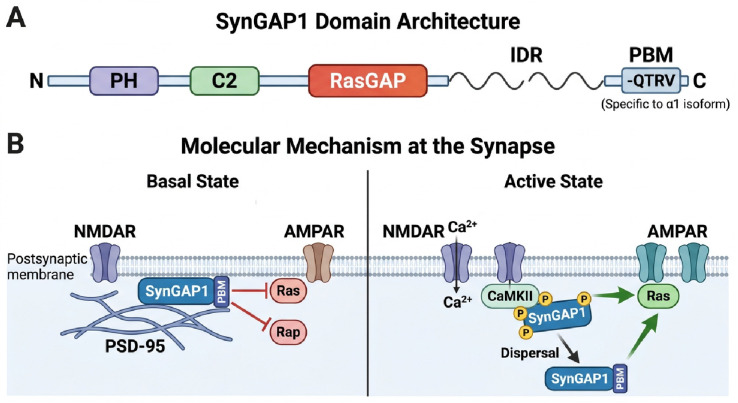
Domain architecture and fundamental molecular mechanisms of SynGAP1. (**A**) SynGAP1 domain architecture. A linear representation of the SynGAP1 protein from the N-terminus to the C-terminus, highlighting the Pleckstrin Homology (PH) domain, the C2 domain, the central GTPase-activating protein (GAP) domain, the intrinsically disordered regions (IDRs), and the PDZ-binding motif (PBM) present in specific isoforms (e.g., α1). (**B**) Molecular mechanism at the synapse. Left: Basal State (Basal inhibition). Under basal conditions, SynGAP1 is anchored to PSD-95 via its PBM. In this state, SynGAP1 exhibits high GAP activity, suppressing Ras/Rap signaling to maintain basal levels of AMPARs. Right: Active State (Synaptic activation and LTP). Upon NMDAR opening, calcium (Ca^2+^) influx into the postsynaptic spine activates CaMKII, which phosphorylates SynGAP1. Phosphorylation causes SynGAP1 to detach from PSD-95 and disperse into the cytoplasm. The relief of SynGAP1-mediated basal inhibition allows robust Ras activation, driving the insertion of new AMPARs to strengthen synaptic transmission, a hallmark of LTP. (Note: PSD-95 remains at the postsynaptic membrane during the active state but is shown detached from SynGAP1 to emphasize the dispersal mechanism). Abbreviations: AMPAR, AMPA receptor; NMDAR, NMDA receptor; LTP, long-term potentiation; CaMKII, calcium/calmodulin-dependent protein kinase II. PSD-95, postsynaptic density protein 95.

To understand this temporal constraint, the dual role of SynGAP1 as a structural scaffold and catalytic enzyme must be examined (Figure 1). The C-terminal QTRV motif specifically binds a multitude of PDZ domains (including, but not limited to, PSD-95), anchoring SynGAP1 into the core synaptic scaffold [16,17,18]. Centrally, the Ras-GAP domain has traditionally been viewed as a catalytic brake on the Ras–extracellular signal-regulated kinase (Ras–ERK) pathways [19,20,21,22]. During synaptic activity, calcium influx and subsequent calcium/calmodulin-dependent protein kinase II (CaMKII) phosphorylation reduce the affinity of SynGAP1 for the PSD scaffold, driving its dispersion to transiently permit downstream signaling [23]. However, as discussed later, the in vivo reliance on this catalytic GAP function versus its physical scaffolding role is currently a subject of intense debate.

### 1.2. The Missing Dimension: Time

Crucially, these mechanisms are temporally orchestrated. Early development relies heavily on structural scaffolding to build connectivity, whereas mature stages increasingly depend on catalytic precision to refine and balance networks. To conceptualize this sequence, we formally define the “Synaptic Clock” as an intrinsic developmental program driven by SynGAP1, functioning in two strictly ordered stages: Phase I (Scaffold Assembly), dedicated to building the physical postsynaptic density through anchoring and phase separation; and Phase II (Catalytic Refinement), where the structural matrix is functionally “loosened” to permit activity-dependent plasticity and precise pathway signaling.

Evaluating existing data through this temporal lens explains that the variable success of rescue experiments is likely tied to the biological phase of the synapse at the time of intervention, rather than merely the chronological age of the organism, providing a theoretical foundation for stage-specific clinical interventions.

## 2. Molecular Mechanisms: SynGAP1 Isoforms and Phase Dynamics

The “Synaptic Clock” is proposed as a biological program driven by specific molecular events. To understand how *SYNGAP1* mutations disrupt neurodevelopment, we must deconstruct the protein’s multidomain architecture and understand how its structural and functional properties are orchestrated over time.

### 2.1. Isoform Switching: The Gearbox of Development

SynGAP1 is a multidomain protein whose core catalytic activity derives from a RasGAP domain, flanked by an N-terminal pleckstrin homology domain, a C2 domain, and extensive intrinsically disordered regions (IDRs) that support multivalent interactions [6,24,25,26]. However, its functional diversity is primarily controlled by alternative splicing, which generates distinct C-terminal isoforms (α1, α2, β, and γ) (Table 1) [16,27,28,29]. Rather than an absolute developmental “on/off switch,” mass spectrometry (MS) data suggest these isoforms are co-expressed at nearly equimolar levels during various developmental windows, with up to 90% of SynGAP1 consistently localized to the PSD across both prenatal and mature stages. Therefore, the developmental timer is likely driven by subtle stoichiometric shifts in their relative ratios rather than wholesale subcellular relocalization.

The α1 isoform, enriched in mature synapses, contains a high-affinity PDZ-binding motif (-QTRV) that stabilizes SynGAP1 within the PSD, promoting persistent Ras suppression and structural maintenance [30,31,32,33]. Its expression surges during a critical period of synaptogenesis and remains high in the mature brain, which is critical for stabilizing the PSD scaffold to maintain dendritic spines and limit AMPA receptor (AMPAR) movement. Conversely, the β and α2 isoforms serve as dynamic regulators. The β isoform lacks the PDZ-binding motif entirely, while α2 harbors a motif with significantly lower PSD-95 affinity [34]. Consequently, these isoforms exhibit greater cytosolic mobility. During early neonatal development, highly expressed β isoforms create a “loosely anchored” synaptic state favoring rapid structural remodeling [27].

Building on these experimentally demonstrated isoform functions, we hypothesize that the shifting α1:β ratio serves as a molecular timer. In this conceptual model, transitioning toward α1 marks the end of the structural assembly phase (Phase I), establishing a stable platform for plasticity. In *SYNGAP1* haploinsufficiency, failing to achieve critical α1 thresholds may leave synapses in a perpetually immature, labile state.

### 2.2. Liquid–Liquid Phase Separation: From Liquid to Gel

SynGAP1’s anchoring role extends to a higher-order biophysical organization: liquid–liquid phase separation (LLPS) [35,36,37]. Through its multivalent coiled-coil domains and IDRs, SynGAP1 assembles with PSD-95 to form dense, membrane-less condensates that concentrate signaling enzymes and receptors. Current in vitro evidence suggests the biophysical state of these condensates evolves developmentally [38,39,40]. In the early “assembly phase,” SynGAP1/PSD-95 condensates exhibit liquid-like properties (behaving much like freely moving oil droplets in water), facilitating the rapid recruitment of components like guanylate kinase-associated protein (GKAP), Shank, and Homer [30]. This dynamism allows flexibility during initial circuit wiring. However, α1 isoform accumulation promotes stronger cross-linking, driving a phase transition toward a stable, gel-like or glassy state. In this context, the transition from “liquid” to “gel” means the local cytoplasm within the spine shifts from a highly dynamic, fluid consistency to a more viscous, heavily cross-linked state, which is thought to be vital for physically stabilizing synaptic receptors.

It is important to note that the current evidence for this biophysical “hardening” is primarily derived from in vitro reconstituted protein systems and primary neuronal cultures. While highly compelling, the exact rheological state of PSD condensates in vivo—and whether a definitive liquid-to-gel transition occurs in the intact brain—remains a subject of ongoing debate. Within the context of our framework, we extrapolate these in vitro findings to propose that this “hardening” is critical for long-term synaptic maintenance. Importantly, this phase behavior is finely regulated by post-translational modifications (PTMs), such as O-GlcNAcylation, which modulates the fusion kinetics of SynGAP1 droplets [41]. We hypothesize that if disrupted by haploinsufficiency, the PSD cannot effectively stabilize AMPARs, potentially undermining the structural foundation required for subsequent catalytic refinement [20,42,43].

### 2.3. Catalytic Gating: Ras, Rap, and the Signal-to-Noise Ratio

While structural anchoring defines Phase I, Phase II involves precise downstream signaling regulation. As a GAP, SynGAP1 exerts tonic inhibition on Ras and Rap1. Under basal conditions, it acts to suppress the Ras–ERK mitogen-activated protein kinase (MAPK) pathway (driving AMPAR insertion) [44,45] and the Rap-p38/phosphoinositide 3-kinase (PI3K) pathways (involved in AMPAR removal and homeostatic scaling) [46,47]. This tonic suppression maintains a high signal-to-noise ratio within the postsynaptic dendritic spine. In this biochemical context, “noise” refers to the unevoked, basal activation of Ras and the spontaneous insertion of AMPARs. By keeping this baseline noise low, SynGAP1 ensures that incoming synaptic stimuli (the “signal”) produce a stark, easily readable molecular burst of Ras activity required for learning. During activity, calcium influx through NMDARs triggers CaMKII, which phosphorylates SynGAP1 (e.g., at Ser1123). This reduces its GAP activity and promotes PSD dispersion, temporarily allowing Ras activation for long-term potentiation (LTP) initiation [27,48,49].

The Synaptic Clock model suggests a developmental hand-off in signaling dominance: Ras regulation is critical early for circuit wiring, while Rap regulation becomes increasingly essential later for synaptic pruning and homeostasis [50,51,52,53,54]. However, it is crucial to acknowledge that this hand-off is likely a relative shift rather than an absolute switch. Literature indicates significant crosstalk between these pathways, with Ras remaining indispensable for adult LTP and Rap functioning in early developmental processes. Thus, these signaling cascades operate as dynamically overlapping networks [27,55].

Crucially, the exact molecular mechanism by which SynGAP1 regulates these overlapping networks is currently under intense re-evaluation. While classically attributed entirely to its enzymatic function, recent compelling in vivo evidence [56] demonstrates that mice carrying mutations that abolish GAP catalytic activity do not exhibit the severe synaptic deficits seen in total haploinsufficiency. This paradigm-shifting data suggests that SynGAP1’s physical scaffolding properties at the PSD may significantly overshadow its enzymatic GAP activity in mature synapses. In this revised context, the downstream “gating” of Phase II might rely heavily on the steric hindrance and spatial organization provided by the massive SynGAP1 protein lattice rather than its pure enzymatic turnover rate. Consequently, in *Syngap1* deficiency, the premature loss of control that leads to a hyper-excitable network and an excitation/inhibition (E/I) imbalance [34] likely stems from the physical collapse of this structural-signaling hub, rather than merely a loss of catalytic enzyme activity.

### 2.4. The Species Gap: Neoteny and the Vulnerability Window

Translating these mechanisms from rodents to humans requires accounting for profound developmental timing differences. Rodents employ a “sprint” strategy, where the α1 isoform switch and PSD-95 stabilization occur rapidly within 2–4 weeks [27,57,58,59], tightly coupling Phase I (assembly) and Phase II (refinement). Humans, however, exhibit pronounced neoteny. Transcriptomic analyses reveal that human *SYNGAP1* upregulation and the α1 shift continue into mid-adolescence (10–15 years) [29,60]. This prolonged timeline provides an extended plasticity window but creates a decade-long vulnerability period. A human *SYNGAP1* defect initiates a cumulative Synaptic Clock misalignment. While this extended gap implies the therapeutic window for structural rescue might theoretically remain open longer in humans than in mice, the downstream effects of this “phase misalignment”—such as altered network wiring—can become far more deeply entrenched over time.

## 3. The Synaptic Clock Framework

### 3.1. Concept Definition: The Imperative of Temporal Sequence

The “Synaptic Clock” model proposes that SynGAP1’s diverse functions represent distinct phases of a unified developmental program. A core proposed principle is sequential coupling: a synapse should ideally achieve structural sufficiency in Phase I before safely supporting the catalytic loosening required for Phase II functional refinement. This model reframes SynGAP1 not merely as a static inhibitor of excitability, but as a dynamic temporal gatekeeper.

Recent studies on patient-specific variants support this temporal separation. While haploinsufficiency uniformly reduces all functions, certain point mutations selectively abolish catalytic activity without disrupting structural stability, and vice versa [57]. This genetic dissociation proves that SynGAP1’s structural and catalytic domains can operate independently. The Synaptic Clock framework extends this spatial modularity into the temporal dimension, positing that healthy development requires these modules to activate in sequence: the structural scaffold assembly of Phase 0/I should ideally be established before the catalytic signaling refinement of Phase II is fully engaged.

Consequently, neurodevelopmental pathology may stem from phase misalignment [61]. If Phase II mechanisms (pruning, homeostatic scaling) begin on a synapse that failed to complete Phase I structural assembly, the network is attempting to refine an inherently unstable structural foundation. This is hypothesized to cause aberrant wiring and circuit hyperexcitability.

### 3.2. Phase Structure and Biological Mapping

**Figure 2:**
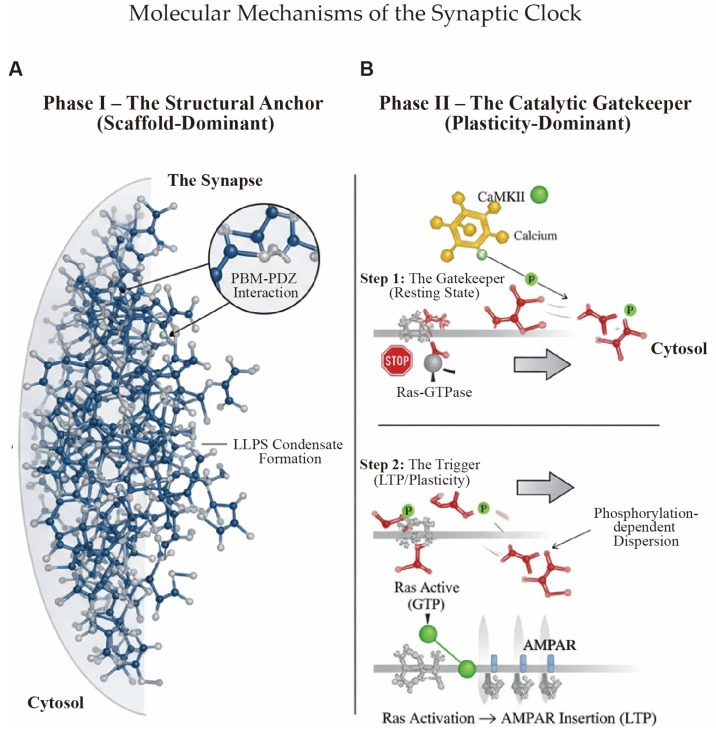
Schematic representation of the proposed hypothesis outlining molecular mechanisms across developmental phases. (**A**) Phase I (Structural Anchor): The α1 isoform (blue) contains a C-terminal PBM. It binds specifically to PSD-95, driving LLPS. This high-density “liquid” state recruits synaptic components, establishing the physical synapse. (**B**) Phase II (Catalytic Gatekeeper): The β isoform (red) lacks the PBM and is less structurally stable. Upon NMDAR activation, CaMKII (yellow) phosphorylates SynGAP1 (an established in vitro and in vivo mechanism). In our model, this causes a hypothesized “phase transition” where SynGAP1 disperses from the PSD, temporarily releasing the brake on Ras to trigger downstream signaling for LTP. Arrows indicate the sequence of molecular events. Abbreviations: CaMKII, calcium/calmodulin-dependent protein kinase II; LLPS, liquid-liquid phase separation; LTP, long-term potentiation; NMDAR, NMDA receptor; PBM, PDZ-binding motif; PSD, postsynaptic density; PSD-95, postsynaptic density protein 95.

Conceptualizing the “Synaptic Clock” as a continuum expands the pathological window of SynGAP1 back to early cortical formation (Figure 2).

To effectively utilize this framework, we propose operational definitions for the biological boundaries that separate these phases. This timeline begins with Phase 0: Cortical Lamination, a pre-synaptic period. Emerging evidence from human cortical organoids suggests SynGAP1 is crucial for radial glia integrity and excitatory neuron migration [62]. However, a recent in vivo study highlights that these Phase 0 neurogenic deficits are absent in SynGAP1 haploinsufficient mice, suggesting that the early role of SynGAP1 in neural progenitor proliferation may be highly species-specific [63]. Operationally, the boundary transitioning from Phase 0 to Phase I is defined by the completion of neuronal migration and the initiation of active synaptogenesis, where its core synaptic functions universally converge across species.

Once neurons assume their proper positions, the clock advances to Phase I: Scaffold Assembly. Molecularly, this is driven by a surge in the SynGAP1 α1 isoform, whose high-affinity PDZ-binding motif anchors to PSD-95 to stabilize the PSD [16,33,64,65]. This anchoring stabilizes F-actin within the dendritic spine head and organizes AMPAR nanoclusters into fixed arrays [66]. Biomechanically, the PSD is held in a highly stabilized state; SynGAP1 strongly inhibits Ras and Rap GTPases, protecting the nascent scaffold from premature, activity-dependent remodeling [14,67].

Only after this structural foundation is secured is the clock proposed to transition to Phase II: Catalytic Refinement, entailing a controlled “loosening” of the architecture to precisely tune neural circuits. Triggered by activity-dependent post-translational modifications (e.g., CaMKII phosphorylation), SynGAP1’s scaffold affinity and GAP activity transiently decrease. In a healthy system, this functional flexibility occurs atop the Phase I platform, ensuring plasticity drives refinement rather than structural instability. Functionally, this critical Phase I-to-II boundary is theorized to be defined by three milestones: the stabilization of the α1:β isoform ratio, the functional establishment of E/I balance (typically following GABAergic shift), and the developmental onset of sensory critical periods.

### 3.3. The Fourth Dimension: Neoteny and Species-Specific Timing

Crucially, the Synaptic Clock scales with species-specific timelines (evolutionary neoteny). In mice, the Phase I-to-II transition happens quickly within weeks. Humans, however, possess a much longer period of cortical maturation. Gene expression data show Phase I molecular signatures (high plasticity, specific isoform ratios) persisting well into human adolescence [29,60]. Therefore, the framework argues that theoretically, clinical interventions should be evaluated based on the patient’s biological “phase status” rather than their chronological age alone.

## 4. A Phase-Stratified Map for SynGAP1 Intervention

**Figure 3:**
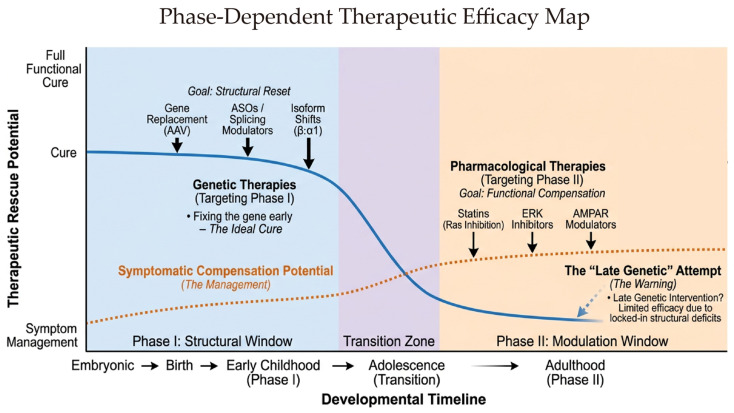
Theoretical synaptic clock therapeutic map. This schematic illustrates the hypothesized relationship between developmental timing and therapeutic outcome. The Blue Solid Curve indicates that genetic interventions aiming to restore the synaptic scaffold are most effective during Phase I. As the brain passes through the critical transition, the potential for a full structural reset is hypothesized to decline. The Orange Dotted Curve represents pharmacological strategies that modulate downstream signaling. These remain effective into Phase II but offer symptomatic management rather than a curative reset. Solid arrows indicate the targeted timing and specific efficacy curves for corresponding interventions, whereas the dashed arrow illustrates the limited efficacy of late genetic intervention. Vertical lines denote the boundaries between different developmental phases. Abbreviations: AAV, adeno-associated virus; AMPAR, AMPA receptor; ASO, antisense oligonucleotide; ERK, extracellular signal-regulated kinase.

The Synaptic Clock framework suggests a shift toward a phase-matched intervention model. By mapping treatments to specific developmental windows, we propose a theoretical roadmap to distinguish strategies that repair synaptic structure from those that modulate functional deficits. This stratified map (summarized in Figure 3 and Table 2) integrates biological rationale with translational risk.

### 4.1. Phase I: Structural Reset via Gene Supplementation

For early-stage Phase I, the theoretical objective is a structural reset. Reinstating the full-length protein during this window could potentially rescue both scaffold assembly and subsequent catalytic regulation.

Preclinical data strongly support this phase-dependent efficacy. In neonatal mice with *Syngap1* haploinsufficiency, viral re-expression successfully normalizes α1 enrichment, rescues dendritic spine morphology, and restores cognitive function [57,68,69,70]. Conversely, intervention delivered after Phase II begins (e.g., in adult mice) yields divergent results: electrical synchrony may improve, but underlying structural deficits in spine density typically persist. This temporal discrepancy aligns with the hypothesis that once the window for early scaffold assembly closes, the potential for a complete structural cure diminishes. Translating this clinically presents challenges, primarily because the large *SYNGAP1* coding sequence (~3.9 kb) pushes the packaging limits of standard adeno-associated virus (AAV) vectors.

### 4.2. The Transition Between Phase I and Phase II: Aligning and Strengthening via Splicing Modulation

As development progresses toward the Phase I–II transition, experimental therapies focus on reinforcing the synaptic scaffold. Antisense oligonucleotides (ASOs) or splice-switching oligonucleotides (SSOs) can be designed to artificially favor α1 production, effectively “strengthening” the synapse even when total protein levels remain haploinsufficient [59,71,72]. Recent progress in human induced pluripotent stem cell (iPSC)-derived neurons demonstrates that SSOs successfully shift isoform ratios and normalize synaptic transmission [73]. However, from a translational perspective, driving excessive α1 enrichment carries significant biological risks. Because mature synaptic plasticity relies on the dynamic dispersal of SynGAP1, an artificial over-accumulation of the α1 isoform could theoretically lock the PSD in an overly stabilized state, potentially impairing cognitive flexibility in older patients.

### 4.3. Phase II: Catalytic Refinement and Pathway Modulation

For models that have entered Phase II, experimental pharmacological agents targeting the Ras/ERK and Rap signaling axes (e.g., MAPK/ERK kinase [MEK] inhibitors) have been explored to correct theorized catalytic imbalance [74]. However, aligned with recent evidence minimizing the isolated role of the GAP domain, treatments based solely on ERK or RasGAP activity modulation have shown mixed or limited translational success in preclinical models. The current line of thought is shifting away from downstream kinase inhibitors, reinforcing the imperative for upstream, protein-restoring therapies (like ASOs) regardless of the developmental phase.

### 4.4. Late Phase II: Compensatory Neuromodulation

In late Phase II, *SYNGAP1* haploinsufficiency causes chronic desynchronization—a failure in the coordinated electrical firing of large populations of neurons—including reduced gamma power and disrupted phase–amplitude coupling, often manifesting clinically on Electroencephalography (EEG) as abnormal background activity or hypersynchronous epileptic discharges. Neuromodulation techniques, such as closed-loop transcranial stimulation, offer a non-invasive method to artificially “pace” these networks [14,78,79,80,81,82]. This strategy does not repair the molecular defect but bypasses it to theoretically restore functional output by recalibrating network timing.

## 5. Biomarkers: Reading the Clock

**Figure 4:**
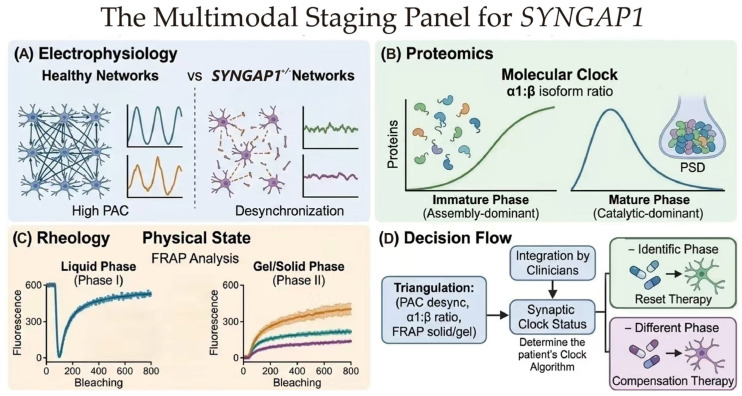
A multimodal biomarker panel for staging *SYNGAP1* status. (Conceptual illustration created by the authors to summarize the proposed framework). To accurately target therapy, the developmental phase must be determined using a triangulation of metrics. (**A**) Electrophysiology: Phase–Amplitude Coupling (PAC) measures the synchronization of neural circuits. Healthy networks show high PAC; *SYNGAP1*-deficient networks show desynchronization (desync). (**B**) Proteomics: The α1:β isoform ratio serves as the molecular clock. A high ratio (green line) indicates an immature, assembly-dominant phase; a low ratio (blue line) indicates a mature, catalytic phase. (**C**) Rheology: Fluorescence recovery after photobleaching (FRAP) analysis reveals the physical state of the PSD. Rapid fluorescence recovery indicates a liquid phase (Phase I), while limited recovery indicates a gel/solid phase (Phase II). (**D**) Decision Flow: By integrating these three metrics, clinicians can determine the patient’s “Synaptic Clock” status and select the appropriate therapeutic modality (Reset vs. Compensation).

Accurate placement of a patient within the Synaptic Clock could be essential for stratified therapy. We propose that biomarkers could act to translate molecular and circuit dynamics into readable clinical signals (Figure 4).

### 5.1. Circuit Signatures: Gamma Oscillations as a Proxy for Phase Integrity

A promising mesoscale readout for *SYNGAP1* deficiency is gamma-band oscillations (30–80 Hz). Both *Syngap1* haploinsufficient mice and human patients consistently exhibit reduced gamma power and disrupted phase–amplitude coupling [83,84,85,86,87,88,89,90]. These gamma oscillations can be recorded directly via local field potentials (LFPs) in animal models, or non-invasively via EEG and magnetoencephalography (MEG) in human patients. This “gamma desynchrony” is interpreted as a functional footprint of the underlying scaffold pathology. However, the field must track the longitudinal trajectory of gamma maturation to properly utilize this metric.

### 5.2. Molecular Staging: From Isoform Stoichiometry to Rheology

We propose a conceptual “Staging Panel” to evaluate the physical state of the PSD (Table 3). The isoform stoichiometric ratio (α1:β) serves as a primary molecular chronometer. Concurrently, experimental Fluorescence Recovery After Photobleaching (FRAP) assays can distinguish dynamic in vitro scaffolds from mature condensates.

### 5.3. The Imperative for Longitudinal Natural History Cohorts

Integrating these biomarkers highlights a critical gap in translational neuroscience: the lack of high-resolution, longitudinal natural history data for *SYNGAP1*-related disorders. Existing cross-sectional datasets often aggregate patients of different ages, inadvertently “blurring” the progression of the disease. To fully harness developmental concepts like the Synaptic Clock for clinical trials, the field urgently requires longitudinal cohorts tracking patients from infancy through adolescence using standardized, multimodal assessments (e.g., longitudinal EEG trajectories, advanced neuroimaging, and standardized behavioral phenotyping).

Ultimately, this data would enable the creation of normative “synaptic growth charts.” By plotting a patient’s biomarker trajectory against normative curves, clinicians could hypothetically identify specific developmental deviations—such as prolonged structural immaturity (theoretically mapping to delayed Phase I assembly) or impaired circuit refinement. Shifting from static, single-timepoint diagnosis to dynamic trajectory tracking represents a critical future direction. This approach will be essential to translate temporal models into practical clinical trial designs, ensuring that disease-modifying therapies (such as ASOs) are evaluated within their optimal, biologically matched developmental windows.

## 6. Challenges and Future Directions: Calibrating the Human Clock

### 6.1. The Delivery Paradox and Biological Risks

The most pressing challenge is the “delivery paradox.” While full structural rescue requires early intervention, the large *SYNGAP1* gene complicates widespread brain delivery. Furthermore, “phase mismatch” poses severe biological risks. For example, administering MEK inhibitors to a neonate during early Phase I to suppress epileptic activity could be developmentally disastrous, as early synaptogenesis strictly depends on a baseline level of Ras-driven trophic signaling [91,92,93].

### 6.2. Regulatory Lag and Resolving Mechanistic Controversies

Translating this accurately requires addressing human neoteny; a therapeutic window lasting weeks in rodents may extend for years in humans, making cross-species calibration a critical unmet need.

### 6.3. Mapping the Human Trajectory

To transform the Synaptic Clock into a viable clinical tool, we must urgently calibrate it for the human cortex, utilizing longitudinally tracked patient-derived iPSC organoids and “bridge studies” with EEG and fluid proteomics.

### 6.4. Stratified Medicine for Broader Chronopathologies

Translational infrastructure must also evolve to support phase-matched adaptive trials, utilizing integrated multi-modal data.

### 6.5. Limitations of the Framework

While the Synaptic Clock offers an integrative perspective, several limitations—many of which reflect active controversies in the field—must be acknowledged:

(1) Isoform Dynamics vs. Absolutes: While our model uses the α1:β ratio as a theoretical timer, quantitative MS data reveal that multiple isoforms coexist at equimolar levels at the PSD throughout development. The “clock” likely relies on subtle, localized micro-domain shifts rather than massive subcellular relocalization.

(2) The Scaffolding vs. Catalytic Debate: A core tenet of classical SynGAP1 biology is its GAP-mediated inhibition of Ras. However, recent in vivo mutant models strongly argue that the physical presence of the protein lattice is far more critical than its enzymatic GAP activity. If true, therapies aimed at downstream signaling (e.g., ERK inhibitors) may be fundamentally limited.

(3) PSD Assembly Sequence: While SynGAP1 is a critical cross-linker, DLG family proteins (like PSD-95) accumulate at immature synapses before SynGAP1, acting as the true primary anchor.

(4) In vitro vs. In vivo Biophysics: The liquid-to-gel transition is heavily supported by in vitro LLPS data, but direct in vivo validation in the intact mammalian brain remains elusive.

These gaps highlight the difference between our proposed conceptual framework and fully established clinical reality (summarized in Table 4).

## 7. Conclusions: The Guardian of Synaptic Time

SynGAP1 functions as a critical temporal regulator of the postsynaptic density rather than merely a static structural component. Through its dual roles as a structural anchor and a catalytic gatekeeper, it drives the precise developmental program that transforms a nascent, highly plastic contact into a mature, stable synapse. Consequently, the pathology of *SYNGAP1*-related neurodevelopmental disorders fundamentally represents a profound loss of timing—a developmental desynchronization that leaves neural circuits permanently out of phase.

This developmental “Synaptic Clock” operates on a prolonged maturation-based timescale, distinctly separate from daily circadian rhythms, and establishes the biophysical boundaries for lifelong synaptic plasticity. By characterizing synaptic maturation as a transition from a fluid-like structural assembly to a consolidated scaffold, this framework provides a cohesive model to explain why distinct intracellular signaling pathways dominate at different developmental stages and why genetic structural rescue possesses a strictly finite therapeutic window.

Ultimately, shifting the perspective from a static biochemical deficit to a dynamic trajectory violation fundamentally alters therapeutic strategies. By prioritizing the validation of longitudinal, phase-specific biomarkers—such as isoform stoichiometry and gamma oscillation synchrony—the field can transition from generic age-based treatments toward biologically stratified clinical trials. This paradigm shift ensures that interventions are precisely matched to the intrinsic developmental state of the patient’s brain, paving the way for true precision medicine in disorders of neurodevelopmental timing.

## Figures and Tables

**Table 1:** Overview of primary SynGAP1 C-terminal isoforms and their proposed functions.

Isoform	PDZ-Binding Motif (PBM)	Synaptic Mobility	Experimentally Established Role & Proposed Developmental Function
α1	Yes (High affinity, -QTRV)	Low (strongly anchored to PSD-95)	Enriched in mature synapses. Stabilizes the PSD structure, restricts AMPA receptor mobility, and maintains basal Ras suppression. Proposed driver of “Phase I” assembly [30,31,32,33].
α2	Yes (Lower affinity)	Moderate	Present across development. Contributes to general synaptic function but provides less rigid scaffolding than α1 [34].
β	No	High (cytosolic/mobile)	Relatively enriched in early developmental stages. Creates a dynamic, loosely anchored synaptic state that favors rapid structural remodeling during early wiring [27].
γ	No	Not fully characterized	Detected in expression profiles, but its specific synaptic functions and developmental roles remain largely uncharacterized compared with other isoforms [27,28,29].

**Table 2:** Therapeutic modalities mapped to synaptic phases.

Therapy Type	Specific Agent	Mechanism of Action	Target Phase	Goal	Current Status	References
Gene Therapy	AAV9-*SYNGAP1*	Gene Replacement (Exogenous expression)	Phase I	Structural Reset (Rebuild Scaffold)	Preclinical	[57,68,69,70]
Oligonucleotide	ASOs (e.g., STK-001)	Splicing Modulation/Upregulation	Phase I	Structural Reset (Boost Levels)	Clinical Trials	[59,71,72,73]
Small Molecule	Statins (Lovastatin)	3-hydroxy-3-methylglutaryl coenzyme A (HMG-CoA) Reductase Inhibition (Reduces Ras farnesylation)	Phase II	Functional Modulation (Dampen Ras)	Clinical Trials	[74]
Small Molecule	ERK Inhibitors	Inhibition of downstream MAPK pathway	Phase II	Functional Modulation (Reduce Excitability)	Preclinical	[19,75,76,77]
Small Molecule	AMPAR negative allosteric modulators (NAMs)	Negative Allosteric Modulation of GluA receptors	Phase II	Functional Modulation (E/I Balance)	Preclinical	[74]
Genetic Tool	Isoform Switch	Clustered regularly interspaced short palindromic repeats (CRISPR)-based forcing of α1 expression	Transition	Structural Extension (Delay Gelation)	Experimental	[71,73]

**Table 3:** The “Synaptic Clock” staging checklist.

Dimension	Metric	Phase I Signature (The Liquid Scaffold)	Phase II Signature (The Catalytic Gel)	Validation Status	References
Stoichiometry (Proteomics)	α1:β Isoform Ratio	High (>1.0) Dominance of C-terminal PDZ-binding motifs.	Low (<0.5) Dominance of GAP-only cytosolic isoforms.	Experimentally Validated (Preclinical & Postmortem)	[16,27,28,29,30,31,32,33,60]
Rheology (Biophysics)	FRAP Mobile Fraction	High Recovery (>70%) Indicates liquid-like, dynamic condensate behavior.	Low Recovery (<30%) Indicates gel-like, immobile lattice behavior.	Theoretical/Exploratory (Validated in vitro, exploratory in vivo)	[35,36,37,38,39,40,41]
Synchrony (Electrophysiology)	Gamma PAC & E/I State	Silent/Assembling Low spontaneous activity; active recruitment of receptors.	Active/Tuned Tight Phase–Amplitude Coupling; established E/I balance (or hyperexcitable in disease).	Experimentally Validated (Preclinical & Clinical trials)	[83,84,85,86,87,88,89,90,91]
Structure (Imaging)	PSD Morphology	Amorphous/Droplet Diffuse boundaries; expanding size.	Defined/Perforated Sharp boundaries; nanocluster organization.	Experimentally Validated (Preclinical models)	[14,66]

**Table 4:** The Synaptic Clock: Evidence vs. Unresolved Questions.

Biological Domain	Experimentally Established Evidence (Current Consensus & Recent Data)	Unresolved Questions & Hypotheses Within the Framework
Isoform Stoichiometry vs. Relocalization	Multiple C-terminal isoforms (α1, α2, β, γ) are co-expressed [16,27,28,29]. Mass spectrometry data indicate that up to 90% of total SynGAP1 consistently localizes to the PSD across both pre- and postnatal developmental stages [60].	If mass subcellular relocalization does not occur, do subtle, highly localized stoichiometric shifts (e.g., altering micro-domain α1:β ratios) act as the primary in vivo timer for critical period closure?
Catalytic GAP Activity vs. Physical Scaffolding	Recent in vivo models demonstrating that point mutations abolishing GAP catalytic activity do not produce the severe synaptic structural deficits observed in total *SYNGAP1* haploinsufficiency [56,57].	Is SynGAP1’s regulation of Ras/Rap networks in mature synapses driven primarily by the steric hindrance of its massive physical lattice rather than its pure enzymatic turnover rate?
PSD Assembly & Structural Anchoring Sequence	DLG family proteins (e.g., PSD-95) accumulate at nascent synapses prior to SynGAP1 [1,2,3,4,5]. SynGAP1 subsequently binds various PDZ domains (via its QTRV motif) to cross-link the emerging network [33,64].	Does SynGAP1 drive fundamental PSD assembly (Phase I), or does it primarily function as a secondary structural stabilizer to consolidate the pre-existing DLG foundation?
Biophysics (Liquid–Liquid Phase Separation)	SynGAP1 and PSD-95 readily undergo LLPS in vitro, forming dense condensates [35,36,37,38,39,40,41]. The rheological properties (liquid-like vs. gel-like) of these droplets are modified by isoform ratios and post-translational modifications.	Does a definitive biophysical “liquid-to-gel” transition occur in vivo within the intact mammalian brain? Are rheological alterations causal to disease, or parallel epiphenomena?
Therapeutic Windows & Targeted Modulation	Early postnatal genetic rescue robustly normalizes structural/spine deficits [13,14,15,57,68,69,70]. In adults, structural rescue is limited, and downstream kinase inhibitors (e.g., MEK/ERK pathways) have shown mixed or poor translational success compared with protein restoration [19,74,75,76,77].	Are upstream protein-restoring therapies (e.g., ASOs) fundamentally superior to downstream catalytic modulation across all developmental phases? Can late-stage interventions reopen structural plasticity?

## Data Availability

No new data were created.

## Abbreviations

The following abbreviations are used in this manuscript:

AAV	Adeno-Associated Virus
AMPAR	α-Amino-3-hydroxy-5-Methyl-4-isoxazolepropionic Acid Receptor
ASD	Autism Spectrum Disorder
ASO	Antisense Oligonucleotide
CaMKII	Calcium/Calmodulin-dependent Protein Kinase II
CRISPR	Clustered Regularly Interspaced Short Palindromic Repeats
DLG	Discs Large
E/I	Excitation/Inhibition
EEG	Electroencephalography
ERK	Extracellular signal-Regulated Kinase
FRAP	Fluorescence Recovery After Photobleaching
GAP	GTPase-Activating Protein
GKAP	Guanylate Kinase-Associated Protein
HMG-CoA	3-Hydroxy-3-Methylglutaryl Coenzyme A
IDRs	Intrinsically Disordered Regions
iPSC	induced Pluripotent Stem Cell
LFP	Local Field Potential
LLPS	Liquid–Liquid Phase Separation
LTP	Long-Term Potentiation
MAPK	Mitogen-Activated Protein Kinase
MEG	Magnetoencephalography
MEK	MAPK/ERK kinase
MS	Mass Spectrometry
NAMs	Negative Allosteric Modulators
NMDAR	*N*-methyl-D-aspartate receptor
PAC	Phase–Amplitude Coupling
PBM	PDZ-binding motif
PH	Pleckstrin Homology
PI3K	Phosphoinositide 3-Kinase
PSD	Postsynaptic Density
PTM	Post-Translational Modification
SRID	*SYNGAP1*-Related Intellectual Disability
SSO	Splice-Switching Oligonucleotide
